# Antibiotic therapeutic drug monitoring in intensive care patients treated with different modalities of extracorporeal membrane oxygenation (ECMO) and renal replacement therapy: a prospective, observational single-center study

**DOI:** 10.1186/s13054-020-03397-1

**Published:** 2020-11-25

**Authors:** Dennis Kühn, Carlos Metz, Frederik Seiler, Holger Wehrfritz, Sophie Roth, Mohammad Alqudrah, André Becker, Hendrik Bracht, Stefan Wagenpfeil, Mathias Hoffmann, Robert Bals, Ulrich Hübner, Jürgen Geisel, Philipp M. Lepper, Sören L. Becker

**Affiliations:** 1 Institute of Medical Microbiology and Hygiene, Saarland University, Homburg, Germany; 2 Department of Internal Medicine V – Pneumology, Allergology and Intensive Care Medicine, Saarland University Medical Center, Homburg, Germany; 3 Department of Anaesthesiology and Critical Care Medicine, University Hospital of Ulm, Ulm, Germany; 4 Department of Medical Biometry, Epidemiology and Medical Informatics, Saarland University, Homburg, Germany; 5 Hospital Pharmacy, Saarland University Medical Center, Homburg, Germany; 6 Department of Clinical Chemistry and Laboratory Medicine, Homburg, Germany

**Keywords:** Antibiotics, Bacteremia, Diagnosis, Infection, Multiresistant bacteria, Sepsis, Therapeutic drug monitoring

## Abstract

**Background:**

Effective antimicrobial treatment is key to reduce mortality associated with bacterial sepsis in patients on intensive care units (ICUs). Dose adjustments are often necessary to account for pathophysiological changes or renal replacement therapy. Extracorporeal membrane oxygenation (ECMO) is increasingly being used for the treatment of respiratory and/or cardiac failure. However, it remains unclear whether dose adjustments are necessary to avoid subtherapeutic drug levels in septic patients on ECMO support. Here, we aimed to evaluate and comparatively assess serum concentrations of continuously applied antibiotics in intensive care patients being treated with and without ECMO.

**Methods:**

Between October 2018 and December 2019, we prospectively enrolled patients on a pneumological ICU in southwest Germany who received antibiotic treatment with piperacillin/tazobactam, ceftazidime, meropenem, or linezolid. All antibiotics were applied using continuous infusion, and therapeutic drug monitoring of serum concentrations (expressed as mg/L) was carried out using high-performance liquid chromatography. Target concentrations were defined as fourfold above the minimal inhibitory concentration (MIC) of susceptible bacterial isolates, according to EUCAST breakpoints.

**Results:**

The final cohort comprised 105 ICU patients, of whom 30 were treated with ECMO. ECMO patients were significantly younger (mean age: 47.7 vs. 61.2 years; p < 0.001), required renal replacement therapy more frequently (53.3% vs. 32.0%; p = 0.048) and had an elevated ICU mortality (60.0% vs. 22.7%; p < 0.001). Data on antibiotic serum concentrations derived from 112 measurements among ECMO and 186 measurements from non-ECMO patients showed significantly lower median serum concentrations for piperacillin (32.3 vs. 52.9; p = 0.029) and standard-dose meropenem (15.0 vs. 17.8; p = 0.020) in the ECMO group. We found high rates of insufficient antibiotic serum concentrations below the pre-specified MIC target among ECMO patients (piperacillin: 48% vs. 13% in non-ECMO; linezolid: 35% vs. 15% in non-ECMO), whereas no such difference was observed for ceftazidime and meropenem.

**Conclusions:**

ECMO treatment was associated with significantly reduced serum concentrations of specific antibiotics. Future studies are needed to assess the pharmacokinetic characteristics of antibiotics in ICU patients on ECMO support.

## Introduction

Sepsis and septic shock due to pneumonia and infections at other body sites are major life-threatening events, which are frequent causes of admission to intensive care units (ICUs) for specific treatment. Rapid initiation of an empirical antimicrobial therapy against the most likely pathogens is key to reduce morbidity and mortality in these patients, as delayed treatment was repeatedly demonstrated to be associated with increased mortality [1–3]. The mortality can be further reduced if the antibiotic treatment regimen is adjusted once the causative agent has been identified (targeted treatment) [4, 5].

Due to increasing rates of multiresistant pathogens and the lack of new antimicrobial agents, pharmacokinetic (PK) and pharmacodynamic (PD) properties of existing antibiotics have been investigated in more detail, and differential dosing schemes were investigated. For antibiotics with time-dependent clinical activity, such as beta-lactams, both prolonged infusion (> 4 h per dose) and continuous administration have been proposed to improve treatment efficacy. PK/PD studies suggest for beta-lactam antibiotics that serum concentrations at about 4–6 times above the minimal inhibitory concentration (MIC, expressed in mg/L) of the causative bacterium are desirable, and these should be maintained for a duration of > 60% of the dosing interval [6, 7]. In 2014, the landmark ‘Defining antibiotic levels in intensive care unit patients’ (DALI) trial demonstrated that serum concentration goals were reached more often when continuous application was performed [8]. Two years later, the ‘Beta-lactam infusion in severe sepsis’ (BLISS) study showed a survival benefit when piperacillin and meropenem were applied continuously in patients with septic shock [9]. Insights gained from recent meta-analyses confirmed positive effects of continuous application of these antibiotics, although a mortality benefit was not consistently seen [10, 11].

Continuous application of anti-infective drugs, however, also carries the risk of constant underdosing if the actual serum concentration of the antibiotic is not measured regularly [8]. Scientific guidelines for pneumonia, sepsis and septic shock, including those of the Surviving Sepsis Campaign [12, 13], thus recommend to employ therapeutic drug monitoring (TDM), i.e. a method to determine antibiotic serum concentrations, if continuous application of antibiotics is used. Indeed, many standard dosing regimens were developed based on data from healthy, normal-weight, mostly male individuals, and are thus not representative for most ICU patients [2, 4, 14]. Patients with severe infectious diseases on ICUs frequently have different PK/PD characteristics due to capillary leak, altered volume of distribution or renal and hepatic insufficiency [7, 15, 16].

Extracorporeal membrane oxygenation (ECMO) is increasingly being used as a rescue therapy for severe acute respiratory distress syndrome (ARDS) or severe circulatory failure [17, 18]. Thus far, there are only limited data as to whether ECMO treatment affects PK/PD parameters of ICU patients significantly. Preliminary evidence suggests that ECMO support seems to alter antibiotic serum concentrations, but standard dosing schemes might be sufficient [19, 20]. However, subtherapeutic as well as significantly elevated, side effect-causing serum concentrations of antimicrobial agents have also been reported [21]. Of note, dosing of the oxazolidinone linezolid seems to be particularly challenging in the ECMO setting [22]. Thus far, little evidence-based guidance exists as to whether specific dosing recommendations should be employed in critically ill patients on ECMO support to counterbalance PK alterations caused by the underlying illness or by drug sequestration in the ECMO circuit [23].

## Objectives

We performed a prospective, comparative clinical study to evaluate and assess the median serum concentrations of continuously applied antibiotics in intensive care patients being treated with and without ECMO in one University Medical Center in southwest Germany.

## Methods

### Study design and location

The study was performed as a single-center, prospective, observational study between October 2018 and December 2019. Consecutive patients treated for severe infections with and without ECMO support were recruited on the pneumological ICU of the Saarland University Medical Center in Homburg, southwest Germany. Serum concentrations achieved by continuous application of the following antibiotics were analysed in the study population: piperacillin, ceftazidime, meropenem, and linezolid. Epidemiological and clinical characteristics of study participants were documented in a case report form (CRF), and we assessed median antibiotic serum concentrations and compared these among patients with and without ECMO.

### Inclusion and exclusion criteria

All patients aged ≥ 18 years who were treated for an acute and severe infectious disease with one of the aforementioned antibiotics on the ICU of our hospital during the study period were eligible to be included. Severe infection was defined as an individual with a sequential organ failure assessment (SOFA) score ≥ 2 at admission to the intensive care unit or an increase in the SOFA score of ≥ 2 in 24 h. Exclusion criteria were an age < 18 years, pregnancy, and/or absence of written informed consent.

### Fluid management

A prespecified hemodynamic protocol to standardise the approach to hemodynamically unstable patients was used. Part of the protocol included the prediction of fluid responsiveness and the restriction of deliberate fluid administration in ARDS patients. Generally, a fluid challenge of approx. 5 mL/kg body weight administered as boli of 50 mL volume was given to reach prespecified aims, one being the increase of venous return to increase cardiac output. Mean arterial pressure target is 60–65 mmHg, if physiologic aims are reached. These include, e.g. capillary refill time (i.e. warm periphery), urinary output (≥ 0.5 mL/kg/h) or lactate levels (≤ 2.0 mmol/L). The need for fluid treatment was determined clinically and on an individual patient basis as judged by a staff intensivist.

### Extracorporeal membrane oxygenation (ECMO) and continuous renal replacement therapy (CRRT)

Depending on the clinical indication, patients were either treated with venovenous (vv)ECMO or venoarterial (va)ECMO. Common indications were ARDS, circulatory failure or bridge to lung transplant. The Cardiohelp System (Maquet Cardiopulmonary GmbH; Rastatt, Germany) was used in all patients, combined with either Deltastream HC (XENIOS AG; Heilbronn, Germany) or HICO-Aquatherm 660 (NUFER-MEDICAL; Bern, Switzerland) as thermoregulatory devices. The oxygenator and tubing used was the HLS Set Advanced 7.0 with Bioline Coating (Maquet Cardiopulmonary GmbH; Rastatt, Germany). Oxygenator membranes were allowed to be used for up to 30 days and blood flow could reach up to 7 l per minute. For the oxygenator membrane, polymethylpenten with a surface area of 1.8 m^2^ and a heat-changing surface area of 0.4 m^2^ was used. In patients requiring continuous renal replacement therapy (CRRT), citrate continuous veno-venous hemodialysis (CVVHD) was used throughout the study with a Fresenius mutiFiltrate machine and an Ultraflux1000s filter (Fresenius SE&Co; Bad Homburg, Germany). To estimate flow rates of ECMO and CRRT for statistical analysis, we calculated the mean flow in the 24 h preceding the antibiotic serum concentration determination.

### Antibiotic treatment modalities

The empirical antibiotic therapy was initiated by the responsible ICU clinician following thorough clinical assessment, and microbiology testing results guided targeted treatment adjustments. All cases were discussed once weekly in an interdisciplinary infectious disease round among intensivists, clinical microbiologists and clinical pharmacists. All patients received a loading dose of the selected antibiotic, followed by continuous application. Dosing schemes and serum target concentrations followed previously published recommendations, with slight modifications [14]. For beta-lactam antibiotics, we defined the target serum concentrations as being four times above the susceptibility breakpoint for Enterobacterales, adhering to the clinical breakpoints recommended by the European Committee on Antimicrobial Susceptibility Testing (EUCAST) in the 2019 version, as follows: (i) ceftazidime, resistant > 4 mg/L → target serum concentration: ≥ 16 mg/L; (ii) piperacillin/tazobactam, sensitive if ≤ 8 mg/L → target serum concentration: > 32 mg/L; and (iii) meropenem, sensitive if ≤ 2 mg/L → target serum concentration: > 8 mg/L. Of note, we employed these target concentrations for Enterobacterales and non-fermentative bacteria. To avoid toxicity, the linezolid target concentration was in the range 6.5–12 mg/L, thus 1.6–3 times above the EUCAST breakpoint at 4.0 mg/L.

### Therapeutic drug monitoring of selected antibiotics

Blood samples for TDM were taken twice a week (every Monday and Thursday) as part of the daily routine blood sampling in patients who received antimicrobial agents using continuous infusion. In all patients, the first TDM measurements were taken ≥ 24 h after treatment initiation with continuously applied antibiotics. All specimens were immediately sent via a pneumatic tube system to the local hospital laboratory for further processing. Upon receipt, samples were instantly centrifuged and the serum was frozen at − 80 °C. A high-performance liquid chromatography (HPLC) assay (Chromsystems Instruments & Chemicals GmbH, Gräfelfing, Germany) was performed to quantify serum antibiotic concentrations on an Agilent 1100 series HPLC system (Agilent Technologies Germany GmbH & Co. KG, Waldbronn, Germany). The laboratory participated regularly in internal and external quality assessment activities. When necessary, antibiotic dose adaptations were performed, adhering to a locally developed standardised operating procedure (SOP). While follow-up TDM measurements were performed in such patients undergoing dose adjustments, we excluded them from this analysis, as it was our goal to assess the effect of standard-dose application (adjusted to renal function, if necessary) on ECMO and non-ECMO intensive care patients. Target attainment was defined as an antibiotic serum concentration above or equal to the aforementioned clinical breakpoints for the respective bacterial pathogens. TDM target attainment was determined for each measurement.

### Clinical microbiology procedures

Microbiological sampling was carried out by the treating clinicians, and samples were immediately processed in the local microbiology laboratory, which employed standard agar plate cultures and polymerase chain reaction (PCR) assays. Pathogen identification was carried out using matrix-assisted laser desorption/ionization time-of-flight (MALDI-TOF) mass spectrometry (Bruker Daltonics; Bremen, Germany). Blood cultures were incubated in a BACTEX FX (Becton Dickinson; Heidelberg, Germany) instrument, and antimicrobial susceptibility testing was performed on a VITEK2 (BioMérieux; Marcy L’Étoile, France) and by Etest methods, using EUCAST breakpoints.

### Statistical analysis

We comparatively analysed data for ECMO and non-ECMO patients, and we used SPSS 25.0 (IBM; Amonk, USA) for statistical analysis. For all continuous variables, either the mean value with standard deviation or the median with interquartile ranges were calculated, as appropriate. Discrete variables were expressed as absolute numbers and percentage. A two-sided p value ≤ 0.05 was considered as statistically significant. To assess differences between the study groups, we used Chi-square test or Mann–Whitney-U test, as appropriate, or t-test for two independent samples. To assess the influence of ECMO, renal function, absence or presence of CRRT, organ failure scores and body mass index (BMI) on serum concentrations, we performed multiple linear generalized estimating equation (GEE) analyses. GEE analyses offer an option for longitudinal analyses in different settings, including repeated measure laboratory experiments [24].

## Results

### Patient characteristics

Between 1 October 2018 and 31 December 2019, we prospectively enrolled 105 consecutive patients on one pneumological ICU, of whom 30 individuals (28.6%) were treated with ECMO support. The mean age in the ECMO group was 47.7 ± 13.1 years vs. 61.2 ± 12.3 years in the non-ECMO group (p < 0.001). There were no significant differences for sex, weight, height, body mass index (BMI) and SOFA score at ICU admission between both groups. Patients on ECMO support required CRRT more frequently (53.3% vs. 32.0%, p = 0.048) and had a significantly elevated ICU mortality (60.0% vs. 22.7%, p < 0.001). Detailed characteristics of the study population are displayed in Table 1.

**Table 1:** Epidemiological and clinical characteristics of patients on an intensive care unit in southwest Germany with and without extracorporeal membrane oxygenation (ECMO) treatment

Characteristic	All patients (n = 105)	Patients on ECMO support (n = 30)	Patients without ECMO support (n = 75)	P
*Epidemiology*				
Male sex	66 (62.9%)	20 (66.7%)	46 (61.3%)	0.661
Age (in years)	57.3 ± 13.9	47.7 ± 13.1	61.2 ± 12.3	< 0.001
Body mass index (BMI)	27.7 ± 8.3	28.8 ± 10.0	27.2 ± 7.5	0.482
*Clinical characteristics*				
Sequential organ failure assessment (SOFA) score (average score and range)	7.0 (4–9)	7.4 (5–8)	6.0 (4–9)	0.340
ICU mortality	35 (33.3%)	18 (60.0%)	17 (22.7%)	< 0.001
Median ICU stay until death (in days)	23 (13–36)	32 (22.5–50)	14 (6.75–23.5)	0.001
*Continuous renal replacement therapy (CRRT)*				
Patients requiring CRRT	40 (38.1%)	16 (53.3%)	24 (32.0)	0.048
CRRT blood flow (in ml/min)	101.1 ± 19.6	100.6 ± 18.9	102.8 ± 25.9	0.411
CRRT dialysate flow (in ml/h)	2307.2 ± 536	2486.2 ± 541.6	2101 ± 470.7	< 0.001
*ECMO characteristics*				
Blood flow (in l/min)		3.9 ± 1.1	–	–
Duration of ECMO membrane oxygenator use (days)		12.6 ± 13.7	–	–

Data were obtained during a study on therapeutic drug monitoring of antibiotics, October 2018–December 2019

### Antibiotic serum concentrations in patients with and without ECMO support

**Fig. 1:**
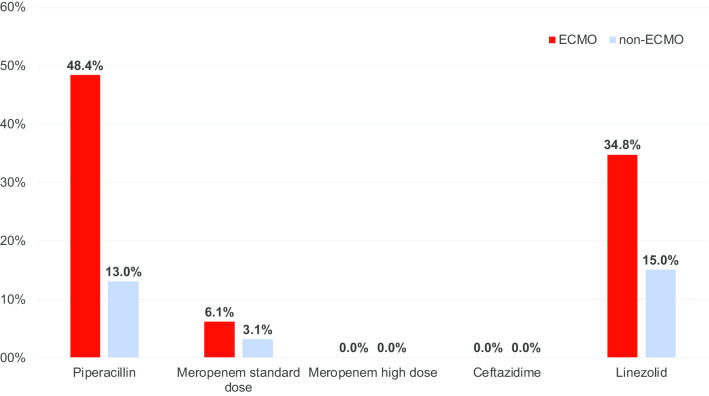
Percentage of intensive care unit patients with and without ECMO support who did not reach pre-specified target serum concentrations (expressed in mg/L) during continuous application of selected antibiotics, southwest Germany, October 2018–December 2019

We analysed 112 antibiotic serum concentration measurements from ECMO and 186 samples from non-ECMO patients, and found significantly lower median serum concentrations for piperacillin (32.3 vs. 52.9; p = 0.029) and standard-dose meropenem (15.0 vs. 17.8; p = 0.020) in the ECMO group. No significant differences were observed for ceftazidime, high-dose meropenem (6 g/d) and linezolid (Table 2). The pre-specified target serum concentrations were not reached for piperacillin and linezolid in 48% and 35%, respectively, of ECMO patients, while this rate was ≤ 15% for both antibiotics in the non-ECMO group (Fig. 1). Using a multiple linear GEE approach to assess the influence of different clinical factors on antibiotic serum concentrations while adjusting for age, sex, body mass index and renal function, we found a statistically significant correlation between ECMO and reduced serum concentrations for piperacillin and standard-dose meropenem. There was no correlation between the blood flow rate on ECMO and decreased antibiotic serum concentrations. Furthermore, the duration of ECMO membrane oxygenator use was associated with an increase in serum concentration of most antibiotics. In contrast to ECMO treatment, CRRT was associated with elevated antibiotic serum concentrations of piperacillin and standard-dose meropenem (Table 3).

**Table 2:** Dosing scheme used for continuous application of selected antibiotics and median serum concentrations of continuously applied piperacillin, ceftazidime, meropenem, and linezolid among patients with and without ECMO support in a pneumological intensive care unit in southwest Germany as compared to pre-defined target serum concentrations, October 2018–December 2019

Antibiotic	Loading dose	Daily dose			Serum target concentration (mg/L)	Patients with ECMO support			Patients without ECMO support			**P**
						Number of patients	Number of TDM measurements	Median serum concentration (mg/L)	Number of patients	Number of TDM measurements	Median serum concentration (mg/L)	
		Normal and moderately impaired renal function	Estimated GFR clearance < 30 ml/min	CRRT								
Ceftazidime	2 g	6000 mg	4000 mg	6000 mg	≥ 16	7	9	49.3 (42.0–69.0)	12	15	63.2 (38.1–71).0	0.69
Piperacillin/tazobactam	4,5 g	13,500 mg	9000 mg	13 500 mg	≥ 32	14	31	32.3 (26.7–55.9)	34	54	52.9 (39.9–87.5)	0.029
Meropenem (standard dose)	2 g	3000 mg (standard dose)	3000 mg	3000 mg	≥ 8	12	33	15.0 (11.8–22.2)	31	64	17.8 (13.4–32.1)	0.020
Meropenem (high dose)	2 g	6000 mg (high dose)	3000 mg	6000 mg	≥ 8	6	16	16.9 (13.7–32.9)	15	33	37.8 (22.6–57.8)	0.372
Linezolid	600 mg	1800 mg	1800 mg	1800 mg	6.5–12	9	23	8.6 (5.0–10.5)	10	20	11.7 (8.3–15.4)	0.618

**Table 3:** Influence of different clinical parameters of intensive care patients treated with ECMO on antibiotic serum concentrations, expressed as p values and determined using multiple linear generalized estimating equation (GEE) analyses in a study from a University medical center in southwest Germany, October 2018–December 2019

Parameter	Ceftazidime	Piperacillin	Meropenem 3 g/d	Meropenem 6 g/d	Linezolid
ECMO	0.69	*0.029*^a^	*0.02*^a^	0.372	0.618
ECMOF24	0.15	0.63	0.86	0.48	0.19
Duration of use of ECMO membrane oxygenator (days)	*0.006*^b^	0.84	*0.014*^b^	0.23	* < 0.05*^b^
CRRT	*0.03*^a^	* < 0.05*^b^	*0.01*^b^	0.36	0.44
CRRT BF24	–	0.556	0.22	* < 0.05*^a^	0.132
CRRT DF24	–	0.2	0.82	*0.01*^a^	0.731

Italic values indicate multiple linear generalized estimating equation (GEE) analyses were used and a p value below 0.05 was considered as statistically significant

The analysis was adjusted for age, sex, body mass index and renal function (expressed as estimated creatine clearance using the CKD-EPI formula). Of note, an average of 3.55 serial measurements of antibiotic serum concentrations were performed per patient (range: 1–14)

*ECMO* extracorporeal membrane oxygenation, *ECMOF24* mean ECMO flow in l/min during 24 h, *CRRT* continuous renal replacement therapy, *CRRT BF24* mean CRRT blood flow in ml/min during 24 h, *CRRT DF24* mean CRRT dialysate flow in ml/h during 24 h

^a^Associated with a decreased antibiotic serum concentration

^b^Associated with an elevated antibiotic serum concentration

### Clinical microbiology findings

A total of 89 potential bacterial pathogens were detected in clinical specimens of 43 patients (17 ECMO patients and 26 non-ECMO patients). Most bacteria were detected in respiratory specimens (50/89, 56.2%), followed by bloodstream infections (26/89, 29.2%) and other foci such as urinary tract or soft tissue infection (13/89, 14.6%). The majority of detected pathogens were Gram-negative bacteria (64/89, 71.9%), with *Escherichia coli* (n = 22), *Pseudomonas aeruginosa* (n = 13), *Klebsiella pneumoniae* (n = 9) and *Enterobacter cloacae* complex (n = 7) being most frequently detected. *Staphylococcus aureus* was the only relevant Gram-positive bacterium recovered from respiratory specimens, whereas coagulase-negative staphylococci and enterococci were mainly detected in blood cultures. Most pathogens were susceptible to the investigated antibiotics, with median MICs below the respective EUCAST breakpoints (Table 4).

**Table 4:** Microbiological findings and minimal inhibitory concentrations (MICs) of antibiotics used to treat bacterial pathogens detected in respiratory tract samples, blood cultures and other body sites of patients in a study on therapeutic drug monitoring of ceftazidime, piperacillin, meropenem and linezolid in southwest Germany, October 2018–December 2019

	Ceftazidime			Piperacillin			Meropenem			Linezolid		
	Median MIC (IQR)	S ≤	R >	Median MIC (IQR)	S ≤	R >	Median MIC (IQR)	S ≤	R >	Median MIC (IQR)	S ≤	R >
*Respiratory tract infection*												
Enterobacterales (n = 27)^a^	1.5 (1–64)	1	4	–	8	16	0.25 (0.25–0.25)	2	8	–	NA	NA
Non-fermenters (n = 15)^b^	2 (1–3)	8	8	136 (16–256)	16	16	0.25 (0.25–0.25)	2	8	–	NA	NA
Gram-positive cocci (n = 8)^c^	–	NA	NA	–	NA	NA	––	NA	NA	2 (2–2)	4	4
*Bloodstream infection*												
Enterobacterales (n = 10)^a^	–	1	4	4 (4–66)	8	16	0.25 (0.25–0.25)	2	8	–	NA	NA
Non-fermenters (n = 3)^b^	–	8	8	4 (4–4)	16	16	–	2	8	–	NA	NA
Gram-positive cocci (n = 13)^c^	–	NA	NA	–	NA	NA	–	NA	NA	2 (1.5–5)	4	4
*Other infections*												
Enterobacterales (n = 10)^a^	1 (1–1)	1	4	4 (4–4)	8	16	0.25 (0.25–1.2)			–	NA	NA
Gram-positive cocci (n = 3)^b^	–	NA	NA	–	NA	NA	-	NA	NA	2 (1–2)	4	4

MIC, minimal inhibitory concentration, expressed as mg/L. IQR, interquartile range. NA, not available

^a^The following Enterobacterales were detected: *Escherichia coli*, *Klebsiella pneumoniae*, *Klebsiella oxytoca*, *Klebsiella aerogenes*, *Proteus mirabilis*, *Enterobacter cloacae* complex, *Hafnia alvei*

^b^The following non-fermentative bacteria were detected: *Pseudomonas aeruginosa*, *Pseudomonas monteilii*, *Achromobacter xylosoxidans*

^c^The folllowing Gram-positive cocci were detected: *Staphylococcus aureus*, *Staphylococcus haemolyticus*, *Staphylococcus epidermidis*, *Enterococcus faecalis*, *Enterococcus faecium*. Of note, only *S. aureus* was considered as clinically relevant in respiratory specimens

## Discussion

The aim of this study was to investigate whether continuous administration of beta-lactam antibiotics and linezolid achieves sufficient serum concentrations in critically ill patients on ECMO support. We found that (i) serum concentrations of piperacillin and standard-dose meropenem were significantly lower in ECMO patients than in non-ECMO patients; and (ii) a considerable amount of ECMO patients treated with piperacillin (48%) and linezolid (35%) did not reach the pre-specified MIC targets. To our knowledge, this is the largest TDM study performed on ECMO patients.

Optimal pharmacotherapy is challenging in critically ill patients whose complex pathophysiological alterations significantly impact on PK/PD characteristics. As antibiotics constitute the decisive causal treatment in severe infections, several large studies were performed to assess the effects of a continuous infusion of beta-lactams. Two randomized trials [8, 9] elucidated that continuous infusion of meropenem and piperacillin were more likely to reach serum concentration goals in critically ill patients than intermittent infusion, and showed that continuous application was associated with a better clinical outcome [25]. Many of these studies recommended a high beta-lactam exposure well above the actual MIC of potential pathogens to account for pathophysiological modifications and the increased risk of multiresistant pathogens on ICUs. For piperacillin/tazobactam, our study confirms previous research that a total daily dose of 13.5 g frequently does not achieve sufficient serum concentrations [26]. However, this dosing regimen (divided in three or four applications per day, or administered as continuous infusion) is still the most widely used on a global scale, and is also endorsed by the Surviving Sepsis Campaign [13], despite accumulating evidence based on clinical experiences and PK/PD modeling simulations that this may be inappropriate. Results from the ongoing multicentric TARGET trial [27], which compares a fixed daily dose of 13.5 g piperacillin/tazobactam to a TDM-guided flexible dosing schedule in ICUs, are likely to document and further substantiate our understanding of the optimal dosing strategy for this drug.

Data pertaining to the continuous application of antibiotics in ECMO patients are scarce, and no specific dosing recommendations have formally been established. A recently published meta-analysis on this topic concluded that the impact of ECMO support on non-lipophilic drugs seems to be negligible [25], which contrasts with our study findings of a significant impact on serum concentrations of piperacillin and meropenem. A TDM study conducted by Donadello et al. on 26 ECMO patients and 41 matched controls did not find significant differences for piperacillin and meropenem serum concentrations between ECMO and non-ECMO patients when intermittent infusion was used. Yet, they reported that > 60% in both patient groups did not reach adequate target concentrations (4–6 times higher than the respective MIC breakpoint). Hanberg and colleagues investigated in another TDM study the effects of intermittent infusion of meropenem on ECMO patients, and showed that a standard dosing regimen (1 g i.v. every 8 h) did not achieve serum concentrations above the MIC of Gram-negative pathogens for the entire dosing interval. Hence, they concluded that alternative dosing strategies might be needed for critically ill ECMO patients with higher odds of multiresistant pathogens. In our study, we found no insufficient target concentrations (i.e., < 8 mg/L) when a higher dose of meropenem (6 g/d) was used.

To our knowledge, our study is the first to perform TDM of continuously applied ceftazidime in ECMO patients. We did not observe a single insufficient serum concentration (< 32 mg/L), which suggests that ceftazidime can safely be used in such patients. Data regarding linezolid in ECMO patients are limited. A case series from Italy used intermittent infusion of 600 mg of linezolid every 12 h in three ECMO patients, which did not achieve clinically effective serum concentrations [28]. Indeed, linezolid dosing on ECMO is challenging [29], and our findings underscore these observations, because we used a higher dose of 1800 mg/d of linezolid for continuous infusion, but still did not reach desirable target serum concentrations in 35% of ECMO patients.

Even though the design of our study does not allow to infer a causal relationship between antibiotic serum concentrations and ECMO, the findings suggest that ECMO support is associated with decreased serum concentrations of piperacillin and standard-dose meropenem. A possible reason for this observation might be sequestration in the ECMO circuit with its large surface. This has been shown for lipophilic and highly protein-bound antibiotics in an ex vivo study and in an in vivo ovine model [30, 31]. Additionally, we found a correlation between increased serums levels of ceftazidime, meropenem and linezolid with a prolonged use of the same ECMO membrane oxygenator. Capillary leak, considerable fluid shifts and the increased volume of distribution in critically ill patients might further contribute to altered serum concentrations of antibiotics. Indeed, ECMO patients are frequently in need of additional fluids to maintain sufficient preload to the extracorporeal system, and commonly have a positive fluid balance [32]. We use a restrictive fluid protocol, yet, it is beyond the scope of this observational study to unambiguously assign the cause for specific antibiotic serum concentrations to patient-specific characteristics or ECMO modalities. Of note, most of the ECMO patients in this cohort were treated with vvECMO for severe ARDS, and half of them required CRRT, which is in line with observations from previous studies [33]. However, our statistical analysis using multiple linear GEE analyses did not provide evidence that the considerable amount of piperacillin or linezolid serum concentrations not reaching the MIC target might have been attributable to CRRT, thus warranting further research on the role of TDM in ECMO settings, with particular emphasis on potential drug sequestration in the ECMO circuit [23].

Several limitations of our study are offered for consideration. First, our assessment was monocentric and causal relationships cannot be inferred. Second, even though the study comprised the largest number of patients in a TDM study of ECMO patients thus far, the overall number is still relatively small. Third, while we considered both renal function and CRRT including specific parameters such as flow rates (e.g. for ultrafiltration), we did not quantify the degrees of e.g. hepatic insufficiency to further characterise potential drug clearance effects, thereby acknowledging that, as in many clinical ICU studies, the profound pathophysiological alterations in critically ill patients cannot be accounted for comprehensively. Previous studies revealed the difficulties to assign PK alterations to either ECMO or CRRT in patients requiring both treatment modalities [19], and further studies aiming at larger patient cohorts are desirable. Fourth, we measured total antibiotic drug serum concentrations instead of free, unbound drug fractions. Hence, as the protein-bound fraction of antibiotics is not clinically active, some of the measured serum concentrations might overestimate the actual amount of effective antibiotic substance in the bloodstream. For example, the protein-bound fraction of piperacillin is estimated at 20–30% of the total serum concentration in intensive care patients [34], and hence, the ‘true’ risk of piperacillin/tazobactam underdosing might be even higher than reported in our study. Indeed, if accounting for a protein-bound fraction of 20% for piperacillin, the resulting serum concentrations would have been insufficient in 22.2% of non-ECMO patients and 54.8% of ECMO patients. If considering a protein-bound fraction of 30%, the rates of subtherapeutic serum concentrations would have been even higher (27.8% in non-ECMO patients vs. 64.5% in ECMO patients). Fifth, it remains to be elucidated whether the employed antibiotics might indeed have been clinically effective in some cases with serum concentrations below the pre-specified target, as the measured pathogen MICs were in most cases well below the respective EUCAST breakpoints (Table 4).

## Conclusions

Our observations suggest that continuous application of beta-lactams and linezolid can be successfully employed in ECMO patients. However, TDM is necessary and should regularly be carried out when piperacillin, standard-dose meropenem and linezolid are administered. Further studies are warranted to assess different dosing regimens for anti-infective drugs in patients on ECMO support, and these should prospectively compare continuous versus intermittent application of selected antibiotics.

## Data Availability

The datasets used and analysed during the current study are available from the corresponding author on reasonable request.

## Abbreviations

ARDS: Acute respiratory distress syndrome
BMI: Body mass index
CRRT: Continuous renal replacement therapy
CVVHD: Continuous veno-venous hemodialysis
ECMO: Extracorporeal membrane oxygenation
EUCAST: European Committee on Antimicrobial Susceptibility Testing
GEE: Generalized estimating equation
HPLC: High-performance liquid chromatography
ICU: Intensive care unit
MALDI-TOF: Matrix-assisted laser desorption/ionization time-of-flight
MIC: Minimal inhibitory concentration
PD: Pharmacodynamic
PK: Pharmacokinetic
PCR: Polymerase chain reaction
TDM: Therapeutic drug monitoring
